# Sera of overweight people promote *in vitro* adipocyte differentiation of bone marrow stromal cells

**DOI:** 10.1186/scrt393

**Published:** 2014-01-09

**Authors:** Giovanni Di Bernardo, Giovanni Messina, Stefania Capasso, Stefania Del Gaudio, Marilena Cipollaro, Gianfranco Peluso, Fiorina Casale, Marcellino Monda, Umberto Galderisi

**Affiliations:** 1 Sbarro Institute for Cancer Research and Molecular Medicine, Center For Biotechnology, Temple University, Philadelphia, PA, USA; 2 Department of Experimental Medicine, Biotechnology and Molecular Biology Section, Second University of Naples, Naples, Italy; 3 IBBR - Institute of Biosciences and Bioresources, CNR, Naples, Italy; 4 Department of Pediatrics “F. Fede”, Second University of Naples, Naples, Italy

## Abstract

**Introduction:**

Overweight status should not be considered merely an aesthetic concern; rather, it can incur health risks since it may trigger a cascade of events that produce further fat tissue through altered levels of circulating signaling molecules.

There have been few studies addressing the effect of overweight status on the physiological functions of stem cells, including mesenchymal stem cells (MSCs), which are the progenitors of adipocytes and osteocytes and are a subset of the bone marrow stromal cell population.

**Methods:**

We decided to investigate the influence of overweight individuals’ sera on *in vitro* MSC proliferation and differentiation.

**Results:**

We observed that *in vitro* incubation of bone marrow stromal cells with the sera of overweight individuals promotes the adipogenic differentiation of MSCs while partially impairing proper osteogenesis.

**Conclusions:**

These results, which represent a pilot study, might suggest that becoming overweight triggers further weight gains by promoting a bias in the differentiation potential of MSCs toward adipogenesis. The circulating factors involved in this phenomenon remain to be determined, since the great majority of the well known pro-inflammatory cytokines and adipocyte-secreted factors we investigated did not show relevant modifications in overweight serum samples compared with controls.

## Introduction

Overweight status and obesity refer to total body weights greater than those considered healthy [1]. Although the public health and medical consequences of the rise in obesity are evident, overweight status is only considered critical for body image and the damaging consequences in terms of health and well-being are often considered negligible in the eyes of the general public as well as specific populations (for example, healthcare professionals) [2]. Nevertheless, several studies have suggested an association between overweight status and human pathologies, such as coronary hearth diseases and chronic kidney disease [3,4].

Studies on the association between overweight status and disease are mainly correlative, and no insights on the biological bases are given. It is evident that in overweight and obese people the level of several circulating cytokines, hormones and other signaling molecules may be dysregulated [4]. This may affect the functions of several organs and tissues, including the stem cell niches, which are subsets of tissues and extracellular subsets that can indefinitely house stem cells and control their self-renewal and progeny production by modulating the concentration of signaling molecules, such as hormones, cytokines, growth factors and so on. [5].

There have been few studies of the effects of overweight status on the physiological functions of stem cells, including those present in bone marrow (BM). The microenvironment of mammalian BM is composed of several different elements that support hematopoiesis and bone homeostasis [6]. It includes a heterogeneous population of cells: macrophages, fibroblasts, adipocytes, osteoprogenitors, endothelial cells and reticular cells. Among these, there are several types of stem cells: hematopoietic stem cells (HSCs), endothelial progenitor cells (EPCs), and mesenchymal stem cells (MSCs), which are a subset of the marrow stromal cell population. MSCs differentiate into mesenchymal tissues, such as bone, cartilage and fat cells, but also support hematopoiesis and contribute to the homeostatic maintenance of many organs and tissues and, therefore, also offer significant therapeutic potential for tissue regeneration. As a result of the multiple roles that MSCs play in the physiology of an organism, impairment of their functions can have profound consequences for body physiology [7-9].

Adipocytes and osteocytes arise from MSCs. Their destinies are not mutually exclusive, but rather are intertwined, as they share a variety of genetic, hormonal and environmental factors. The available literature indicates that obesity may decrease osteoblastogenesis while increasing adipogenesis. It remains to be determined how obesity may affect these processes [10]. Some clues may be derived by considering that the traditional view of adipose tissue as a passive reservoir for energy storage is no longer valid. Indeed, the physiology and role of adipose tissue is more complex than previously thought. Besides the white adipose tissue (WAT), which is the most abundant human fat, there are two other adipose tissue types that differ significantly from WAT. Brown adipose tissue (BAT), the main role of which is the regulation of thermogenesis through burning of energy rather than its storage is the second type. Bone marrow adipose tissue (BMAT) is the third fat depot and has similarities to both WAT and BAT. Fat occupies a significant portion of the bone cavity; however, its role is largely unknown. The BMAT was traditionally thought to have no function and has been overlooked or ignored for a long time [11]. Several studies have shown that cells in the bone marrow niche communicate with each other and are essential for the maturation and correct functioning of MSCs and HSCs. Adipocytes in bone marrow may cooperate with resident stem cells by acting as placeholders until the stem cells differentiate into the cell type that is needed. BMAT may also play a role in energy storage and thermogenesis and impaired functions of BMAT may influence bone remodeling through the secretion of cytokines that target bone, the production of signaling molecules that affect sympathetic impulses to bone and also through the paracrine influences on adjacent skeletal cells [12].

In overweight and obese people, the dysregulated level of circulating signaling factors may also affect the differentiation potential of bone marrow resident MSCs, altering the equilibrium between adipo- and osteogenesis. We decided to investigate this phenomenon by analyzing the influence of sera from overweight individuals on *in vitro* MSC proliferation and differentiation.

## Methods

### Ethical approval

The experimental procedures followed the rules approved by the Ethics Committee of the Second University of Naples. In detail, patients were informed of the research and gave permission for the use of serum samples and/or bone marrow harvests.

### Serum samples

Serum samples were collected from five adult men of healthy weight (body mass index (BMI) <25) and eight adult men with BMIs >25 (overweight), after informed consent. Whole blood samples (10 ml) were collected from patients in Vacutainer test tubes (BD Bioscience, Buccinasco, Italy). After collection, the samples were left undisturbed to allow the blood to clot at room temperature. The clots were removed by centrifuging at 1,000 to 2,000 g for 10 minutes in a refrigerated centrifuge. The resultant supernatants were designated sera and were collected with a Pasteur pipette.

We pooled sera from the healthy weight and overweight samples to create two different experimental groups: ‘healthy weight’ (HS) and ‘overweight’ sera (OS), respectively.

### Bone marrow stromal cell cultures

Bone marrow was obtained from three healthy donors. We used bone marrow from a 10-year-old, 12-year-old and 13-year-old male donor, after their parents gave informed consent. We separated cells using a Ficoll density gradient (GE Healthcare, Milan, Italy), and the mononuclear cell fraction was collected and washed in PBS. We seeded 1 to 2.5 × 10^5^ cells/cm^2^ in alpha-minimum essential medium (alpha-MEM) containing 10% fetal bovine serum (FBS) and 1 ng/ml beta-fibroblast growth factor (β-FGF). After 72 hours, non-adherent cells were discarded, and adherent cells were further cultivated to confluency. Cells were then incubated for seven to ten days in proliferating medium to reach confluence and extensively propagated for our experimental plan. We verified that, under our experimental conditions, the bone marrow stromal cultures contained MSCs that fulfilled the three criteria proposed to define MSCs [13]. All experiments were carried out on MSC cultures at passage 3.

For evaluation of the effects of OS and HS on *in vitro* MSC functions, cells were incubated for 72 hours in alpha-MEM containing 10% human serum pools and 1 ng/ml β-FGF. At the end of that time, samples were collected for analysis.

All cell culture reagents were obtained from Euroclone Life Sciences (Milan, Italy) and Hyclone (UT, Logan, USA) unless otherwise stated.

### Annexin V assay

Apoptotic cells were detected through the use of fluorescein-conjugated Annexin V (Roche, Milan, Italy) following the manufacturer’s instructions. Apoptotic cells were observed through a fluorescence microscope (Leica Italia, Milan, Italy). In every experiment, at least 1,000 cells were counted in different fields to calculate the percentage of dead cells in each culture.

### Senescence-associated β-galactosidase assay

Cells were fixed for 10 minutes with a solution of 2% (v/v) formaldehyde and 0.2% (w/v) glutaraldehyde. Cells were washed with PBS and then incubated at 37°C for at least two hours with a staining solution (30 mM citric acid/phosphate buffer (pH 6), 5 mM K_4_Fe(CN)_6_, 5 mM K_3_Fe(CN)_6_, 150 mM NaCl, 2 mM MgCl_2_, 1 mg/ml 5-bromo-4-chloro-3-indolyl-beta-D-galacto-pyranoside (X-Gal) solution). The percentage of senescent cells was calculated by the number of blue cells (β-galactosidase positive cells) out of at least 500 cells in different microscope fields.

### Adipogenic differentiation

Bone marrow stromal cultures were incubated for 72 hours in alpha-MEM containing 10% of each serum pool (HS or OS) and β-FGF. Then the cells were stimulated for 15 days in hMSC mesenchymal stem cell adipogenic differentiation medium (catalog n. PT-3004-KT - Lonza, Walkersville, MD, USA). The medium contains insulin (recombinant), dexamethasone, indomethacin and 3-isobuty-l-methyl-xanthine (IBMX). Lipid droplets were revealed by staining with Oil Red O. Adipogenic differentiation was evaluated by determining the expression of genes involved in adipogenesis, such as C/EBPß and C/EBP∂ (early genes) and PPARγ, C/EBPα, LPL and ATGL (late genes).

### Osteogenic differentiation

After 72 hours of serum treatment, HS or OS cells were stimulated for 15 days in hMSC mesenchymal stem cell osteogenic differentiation medium (catalog n. PT-3002-KT-Lonza). The medium contains dexamethasone, ascorbate and glycerophosphate. Staining with Alizarin red revealed calcium deposits in differentiated osteocytes. Osteogenic differentiation was evaluated by determining the expression levels of osteopontin and osterix, both involved in osteogenesis.

### Reactive oxygen species detection

For each serum group (HS or OS), intracellular reactive oxygen species (ROS) levels were investigated using the d-ROMs test (Diacon, Grosseto, Italy) according to the manufacturer’s instructions. ROMs (hydroperoxides, ROOH, primarily) in a biological sample in the presence of iron (released from plasma proteins by an acidic buffer, the R2 reagent in the kit) are able to generate alkoxyl (R-O*) and peroxyl (R-OO*) radicals, through the Fenton reaction. Such radicals, in turn, are able to oxidize an alkyl-substituted aromatic amine (A-NH2, solubilized in a chromogenic mixture, the R1 reagent of the kit), thus transforming them into a pink-colored derivative ((A-NH2*)+). Finally, this colored derivative is photometrically quantified by measuring absorbance (at 505 nm or 546 nm) (Tecan, Mannedorf, Switzerland). The intensity of the developed color is directly proportional to the concentration of ROMs, according to Lambert-Beer’s law.

### Cytokine array

The profile of the relative levels of 18 cytokines in the serum samples harvested from the healthy weight and overweight groups was determined using the Human Cytokine Antibody Array 1.0 (Affymetrix, Emeryville, CA, USA). The nitrocellulose membranes provided by the manufacturer contain 18 capture antibodies spotted in duplicate on the surface. Each membrane also included four pairs of positive control spots and two pairs of negative control spots. A total of 2 ml of the serum samples for each of the two experimental groups was used for hybridization. Hybridizations and signal measurements were done following the manufacturer’s instructions. Array signals were acquired using the Chemidoc system (Bio-Rad Company, Hercules, CA, USA) and the associated software QuantityOne. Array images used for signal quantification (expressed as pixel density) were produced through five minute camera exposures. All the membranes were processed simultaneously. All hybridizations were repeated twice.

### RNA extraction and RT-PCR

Total RNA was extracted from the cell cultures using TRI REAGENT (Molecular Research Center Inc., Cincinnati, OH, USA) according to the manufacturer’s protocol. The mRNA levels of the analyzed genes were measured by RT-PCR amplification, as previously reported [14,15].

Sequences for mRNAs from the nucleotide data bank (National Center for Biotechnology Information, Bethesda, MD, USA) were used to design primer pairs for RT-PCR reactions (Primer Express, Applied Biosystems, Carlsbad, CA, USA). Primer sequences are in Additional file 1. Appropriate regions of GAPDH cDNA were used as controls. PCR cycles were adjusted to have linear amplification for all the targets. Each RT-PCR reaction was repeated at least three times. A semi-quantitative analysis of mRNA levels was carried out using the ‘GEL DOC UV SYSTEM (Bio-Rad). Primer sequences were designed with Primer Express software (Invitrogen, Milan, Italy).

### Statistical analysis

Statistical significance was evaluated using analysis of variance (ANOVA) followed by Student’s t and Bonferroni’s tests. In analyzing the data with randomized group design, the variances within and between the groups need to be counted. We used mixed-model variance analysis for data with continuous outcomes. All data were analyzed with GraphPad Prism-version 5.01 statistical software package (GraphPad, La Jolla, CA, USA).

## Results

**Figure 1:**
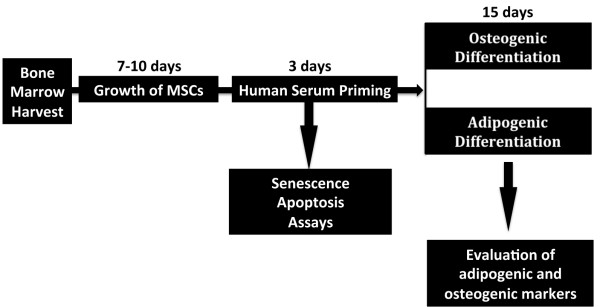
**Experimental plan.** Bone marrow was collected from healthy patients and mononuclear cell fractions were used to provide bone marrow stromal cultures containing MSCs. Cultures were propagated for seven to ten days. Then cultures were treated with OS and HS for three days (priming). At the end of priming, apopotosis and senescence were evaluated. Cultures were then incubated in adipogenic or osteogenic differentiation media for 15 days and the differentiation processes were evaluated. HS, healthy weight sera; MSCs, mesenchymal stem cells; OS, overweight sera.

We divided our sample into two groups: HS (n = 5) and OS (n = 8). We did not observe significant intra- or inter-group differences in the levels of the main blood serum biochemical indicators (Table 1 and Additional file 2). For this reason, we adopted a pooling strategy to compensate for the limited numbers of samples and to reduce biological variation [16]. The overall research strategy is depicted in Figure 1.

**Table 1:** Main blood serum biochemical indicators

**Patient parameters**	**Healthy weight**	**Overweight**
BMI (Kg/m^2^)	21.10 ±1.10	29.63 ± 1.80*
Glucose (mmol/l)	88.8 ± 5.22	90.63 ± 8.94
Total cholesterol (mmol/l)	205.6 ± 26.18	203.5 ± 42.37
LDL cholesterol (mmol/l)	124.8 ± 24.10	131.6 ± 41.27
HDL cholesterol (mmol/l)	65.6 ± 15.14	56.4 ± 8.52
Triglycerides (mmol/l)	77.2 ± 30.43	100.1 ± 46.42

Patients were divided into two groups of healthy weight (n = 5) and overweight (n = 8) individuals, that showed significant differences (*P* <0.05) in BMI. Other parameters did not present statistically significant differences and were in the normal value range for both groups. Data are expressed as mean values with standard deviations (*P <0.05). BMI, body mass index; HDL, high density lipoprotein; LDL, low density lipoprotein.

### Overweight sera did not affect the proliferation, apoptosis or senescence rate of MSC cultures

We evaluated whether some *in vitro* biological properties of MSCs were affected differently by incubation with OS compared with cells treated with HS. Proliferation rates of MSCs incubated with OS did not differ significantly from those treated with HS [see Additional file 3]. Changes in the circulating cytokines and hormones of overweight people may affect the cell biology of MSCs and drive cells to different possible fates, including apoptosis and senescence. These outcomes are not mutually exclusive, despite the fact that some cellular stresses preferentially induce one or the other of these two fates [17].

**Figure 2:**
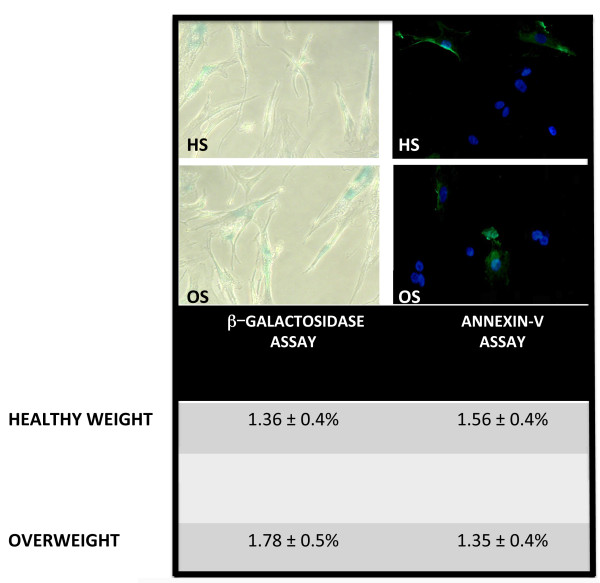
**Senescence and apoptosis assays.** Acid β-galactosidase and Annexin V assays were carried out to detect senescent and apoptotic cells in MSC samples treated with HS and OS. The picture shows representative fields of acid β-galactosidase (left) and Annexin staining (right). Arrowheads indicate senescent cells. Annexin-positive cells are green. Cells were counterstained with DAPI (blue). Mean expression values for senescent and apoptotic cells are indicated in the corresponding table (± SD, number of experiment replicates: three). DAPI, 4',6-diamidino-2-phenylindole; HS, healthy weight sera; MSCs, mesenchymal stem cells; OS, overweight sera.

The Annexin assay did not show a significant difference in the percentage of apoptotic cells in cultures treated with OS as compared to the controls (Figure 2).

The senescence process was also unaffected by OS treatment, as detected by the acid beta-galactosidase assay (Figure 2).

### Adipogenic differentiation

**Figure 3:**
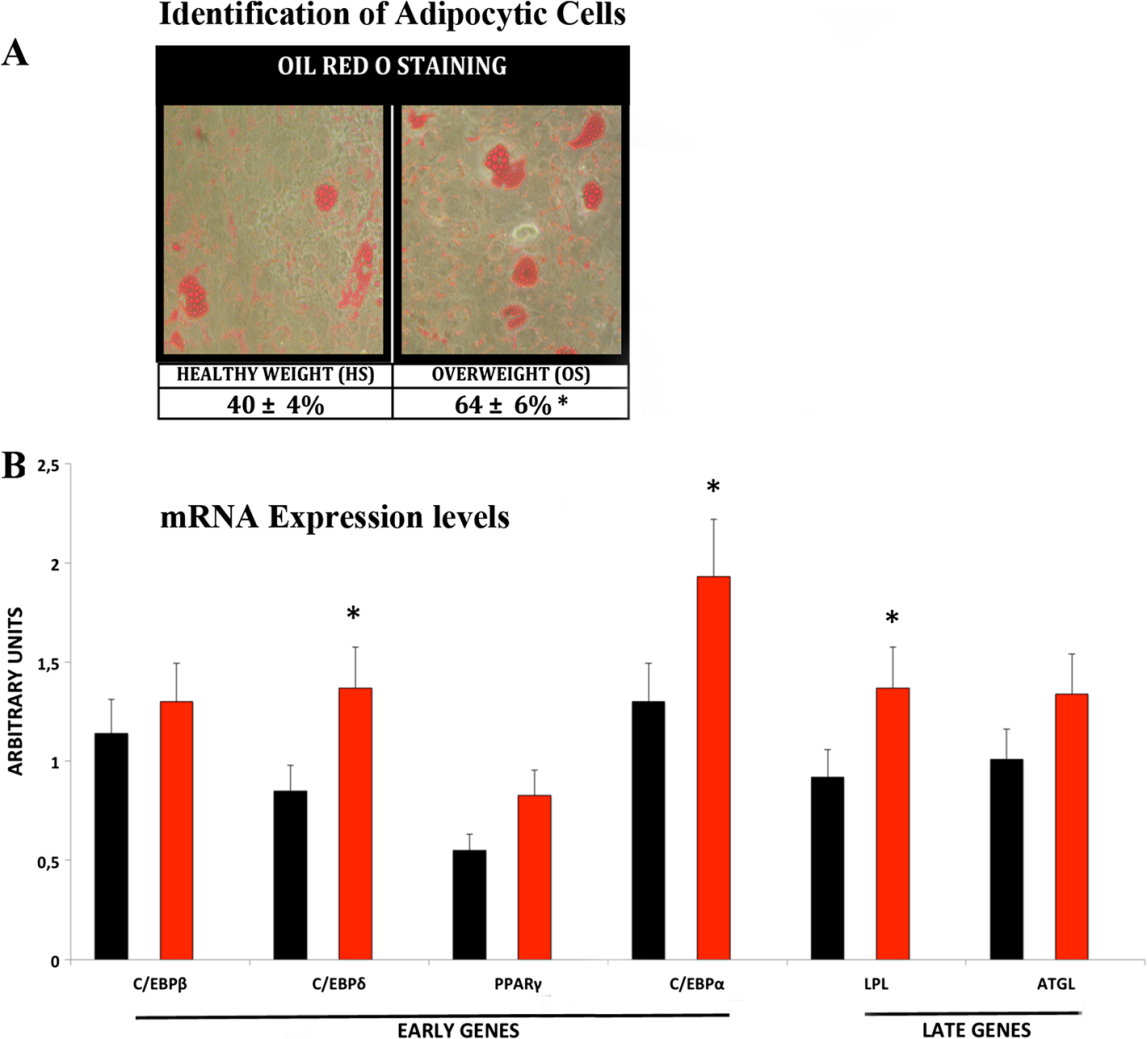
**Analysis of adipocyte differentiation. A)** The table shows the percentage of Oil Red O positive cells treated with OS or HS and then induced to differentiate into adipocytes. The percentage of Oil Red O positive cells was calculated by counting at least 500 cells in different microscope fields. Data are expressed as mean values with standard deviations (*P <0.05). The picture shows a representative field of oil-red positive cells. **B)** RT-PCR expression analysis of early and late adipocyte differentiation markers in MSCs treated with OS or HS and then induced to differentiate into adipocytes. mRNA levels were normalized with respect to GAPDH, which was chosen as an internal control. Each experiment was repeated at least three times. The histogram shows the changes in mRNA expression levels 14 days after incubation in differentiation conditions of MSCs grown in OS (red bars) or HS (black bars). They are expressed as arbitrary units (*P <0.05). HS, healthy weight sera; MSCs, mesenchymal stem cells; OS, overweight sera.

Fat accumulation is closely related to bone formation and resorption, and it has been suggested that obesity may decrease bone formation while increasing adipogenesis [10]. For this reason, we looked at the effects of OS on MSC differentiation into adipocytes. MSC cultures were incubated for 72 hours in alpha-MEM containing 10% of OS or HS. The cells were then stimulated for 15 days in mesenchymal stem cell adipogenic differentiation medium (Lonza). OS treatment induced a higher percentage of differentiated adipocytes (64 ± 6%) compared with HS (40 ± 4%), as determined by Oil Red O staining (Figure 3A). These data were confirmed by expression analysis of early (C/EBPß and C/EBPδ) and late (PPARγ, C/EBPα, LPL, and ATGL) adipocyte differentiation markers. In proliferating MSCs we detected only a minimal amount of C/EBPß and C/EBPδ both in cells grown with HS and with OS; there were no significant differences between the two experimental conditions. Following incubation in differentiation medium, we observed a higher increase in the expression of adipogenic markers in OS treated cultures, compared with cells incubated with HS (Figure 3B).

### Osterix and osteopontin follow up in osteogenic differentiation

**Figure 4:**
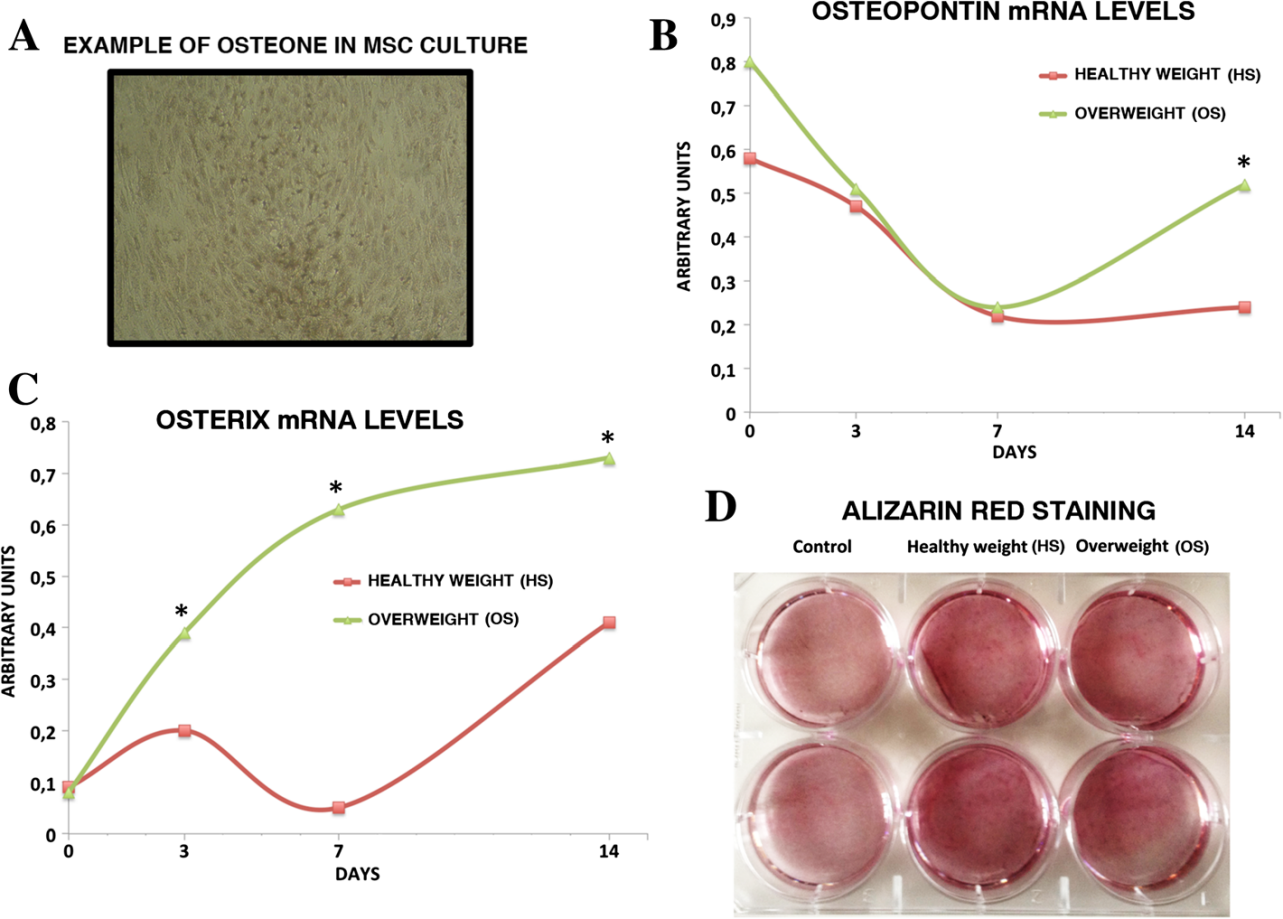
**Analysis of osteocyte differentiation. A)** The picture shows a representative image of an osteon formation following osteocyte differentiation of MSCs. Image taken on an upright inverted microscope with a 20× objective. The graph represents the expression follow up of osteopontin **(B)** and osterix **(C)** during osteocyte differentiation of MSCs treated with OS or HS. mRNA levels were normalized with respect to GAPDH, which was chosen as an internal control. Each experiment was repeated at least three times. The histogram shows the mRNA expression levels. They are expressed as arbitrary units (*P <0.05). **D)** The picture shows Alizarin red staining of MSCs treated with OS or HS and then induced to differentiate into osteocytes. Control: cells not induced to differentiate. The Alizarin red staining intensity for each cell culture dish was acquired with a CCD camera and analyzed with Quantity One 1-D analysis software (Bio-Rad). We calculated the sum of the fluorescent pixel values of stained cells and then determined the average fluorescent pixel intensity. HS, healthy weight sera; MSCs, mesenchymal stem cells; OS, overweight sera.

We examined the effects of OS on MSC differentiation into osteocytes in a similar fashion (Figure 4A, B, C, D). Alizarin red staining did not show considerable differences in the osteogenesis process of MSCs incubated with OS or HS (Figure 4D). To gain further insights into osteocyte differentiation, we performed a follow up expression analysis of osteopontin and osterix, which are involved in the osteocyte differentiation process [18,19].

In HS-treated MSCs, the differentiation marker osterix showed a typical bimodal expression profile, with a burst in expression during the first stage of differentiation (Figure 4C). This expression pattern was altered in the OS-treated MSCs. The osteopontin expression profile was also altered in OS-treated cells compared with HS samples. As expected, in HS-treated MSCs, the expression level of osteopontin, an early differentiation marker, was high in the first days of differentiation, then declined and remained stable during the entire maturation process (Figure 4B). On the contrary, in OS-treated MSCs, osteopontin expression, after an initial decrease, exhibited a progressive increase in mRNA levels during the late differentiation phase (Figure 4B). This result suggests that osteocyte differentiation may be dysregulated in OS samples.

### Comparison of cytokine expression profiles in overweight and healthy weight sera

Adipose tissue secretes a variety of products known as adipokines, including leptin, adiponectin, resistin, and visfatin, as well as cytokines and chemokines such as TNF-α, IL-6, and monocyte chemoattractant protein-1 (MCP-1). The release of adipokines by either adipocytes or adipose tissue-infiltrated macrophages leads to low-grade inflammation, a hallmark characterizing adult obesity, which may be a pivotal mechanism linking obesity to its numerous systemic complications [20].

**Figure 5:**
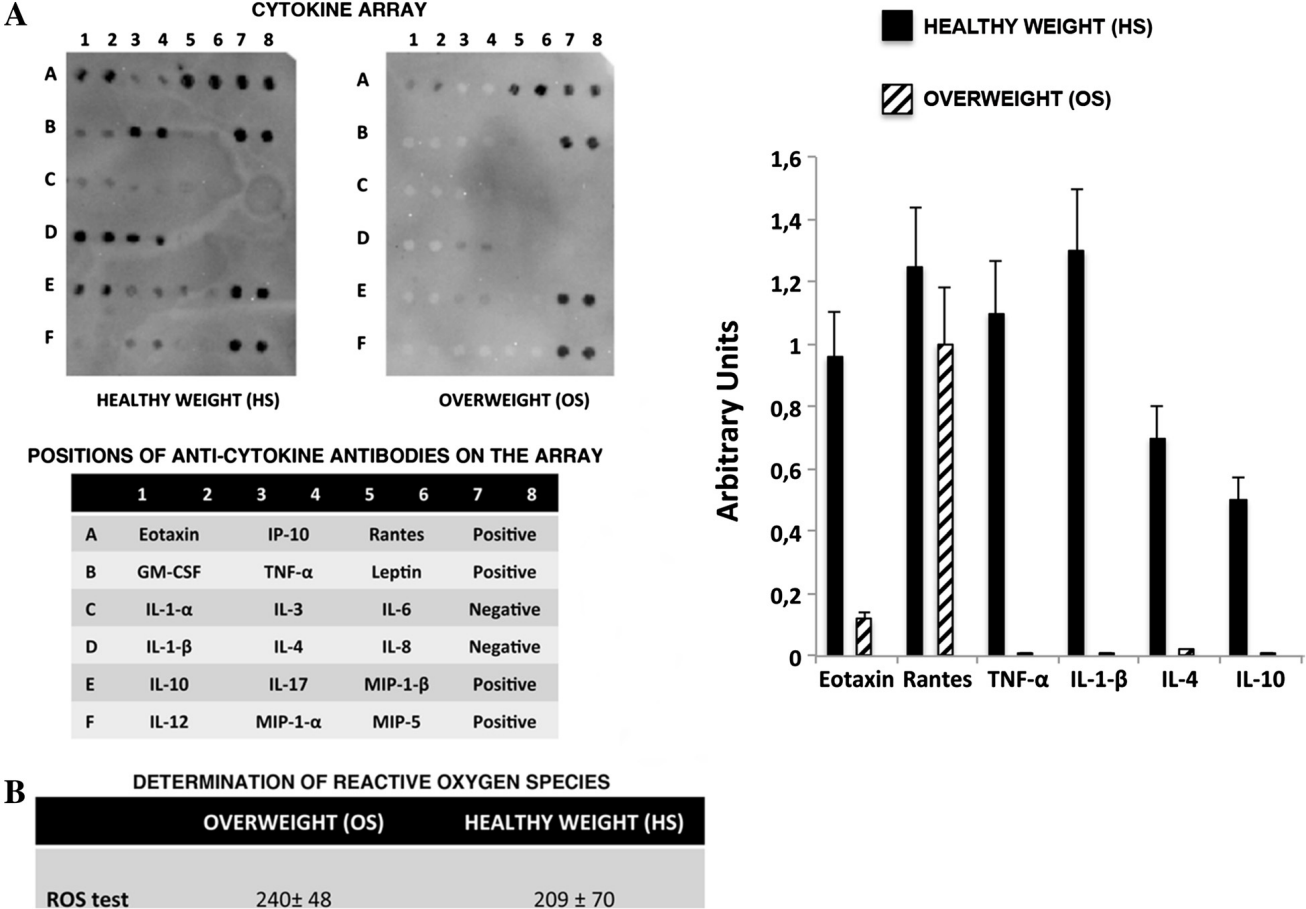
**Cytokine and reactive oxygen species (ROS) detection in sera. A)** Arrays incubated with HS and OS samples. The table below the arrays shows the name and the relative position on the Panomics TranSignal Human Cytokine Antibody Array of the cytokines that were detected in OS and HS sera. On the table ‘Positive’ and ‘Negative’ are the array internal controls. Array signals were acquired using the Chemidoc system (Bio-Rad) and the associated software QuantityOne. The graph shows the cytokine expression levels in the OS and HS sera. Data are expressed as arbitrary units (*P < 0.05). **B)** The table shows the expression of ROS in HS and OS samples. Data are expressed in arbitrary units (± SD, number of experiment replicates: three). HS, healthy weight sera; OS, overweight sera.

We used the Panomics TranSignal Human Cytokine Antibody Array (Affymetrix) to accurately profile the expression of 18 of the most studied cytokines. The expression levels of several cytokines did not differ significantly between the OS and HS samples. Several cytokines were easily detectable on the arrays but their low levels did not allow a quantitative comparison (Figure 5A). Notably, levels of leptin, whose synthesis and secretion is increased in obese subjects in proportion to the degree of adiposity, did not differ significantly in overweight samples compared with controls (Figure 5A) [21].

Several findings support a direct correlation between the levels of inflammatory cytokines (IL-1β, IL-6, TNF-α) and BMI [22,23]. Unexpectedly, TNF-α and IL-1β levels were lower in the OS than the HS, while no significant modification of IL-6 was detected (Figure 5A) [24]. In OS we also observed a decrease in the expression of the anti-inflammatory cytokine IL-10 (Figure 5A).

Fat accumulation is correlated with systemic oxidative stress in humans and mice. Production of ROS increases selectively in the adipose tissue of obese mice, accompanied by augmented expression of NADPH oxidase and decreased expression of antioxidative enzymes [25]. We decided to investigate if an increased level of ROS in OS may account for its effect on adipogenesis, since there are reports showing that increases in intracellular ROS levels mediate the adipocytic differentiation of MSCs [26]. The ROS levels in sera from OS and HS samples did not differ significantly as detected by the d-ROMs test (Diacon) (Figure 5B).

## Discussion

The great majority of studies on obesity focus on the analysis of wholly obese individuals (BMI >30). Nevertheless, it is becoming clear that overweight status should be considered not merely an aesthetic concern, but rather can also be related to health risks. In addition, overweight status may trigger a cascade of events, increasing fat tissue through alterations in levels of circulating signaling molecules. We decided to investigate the effect of sera from overweight individuals on the differentiation potential of MSCs, which are precursors of adipocytes and osteocytes. This represents a pilot study due to the small number of cases evaluated. For this reason, the conclusions based on this finding must be confirmed with further analytical studies employing the sera of a larger number of subjects.

Several reports support the existence of a homeostatic system that dynamically adjusts energy intake and energy expenditure to promote body fat mass stability. In the context of this system, the ease with which many individuals gain weight is difficult to explain. Our data may offer a hint about the mechanism underlying these observations. We observed that *in vitro* incubation of MSCs with the sera of overweight individuals promotes adipocytic differentiation of MSCs, while partially impairing proper osteocyte differentiation (Figures 3 and 4). These results may suggest that becoming overweight triggers a further gain of weight by promoting a bias in the differentiation potential of MSCs toward adipogenesis. This may occur through a modification in the expression pattern of circulating cytokines, hormones, and growth factors. This hypothesis is in agreement with the theory that energy homeostasis operates primarily to defend against weight loss and that, over the course of evolution, biological defense against weight gain was not selected for [27].

The circulating factors involved in this phenomenon remain to be determined, since the great majority of the well-known pro-inflammatory cytokines and adipocyte-secreted factors we investigated did not show relevant modifications in OS samples compared with controls.

It now appears that in most obese patients obesity is associated with a low-grade inflammation, resulting from chronic activation of the innate immune system and an increase in circulating pro-inflammatory cytokines [28].

Indeed, several studies have demonstrated a direct correlation between the levels of inflammatory cytokines (IL-1β, IL-6, TNF-α) and BMI. Nevertheless, our data conflict with these previously published data, in that serum IL-6 levels were similar between overweight and control groups, and TNF-α and IL-1β levels were lower in overweight subjects, although others have shown no elevation of serum IL-6 or TNF-α in obese patients [24,29,30].

There are some potential explanations for these discordant data. The main hypothesis is that our analysis was carried out on overweight individuals, so alterations in circulating cytokines may not be strictly comparable with those of obese subjects. It should be emphasized that in overweight samples we detected a decrease of IL-10, an anti-inflammation cytokine. This may suggest that in overweight individuals the pro-inflammation status may be sustained by changes in cytokine expression that do not completely overlap with those observed in obese subjects.

Larger studies in which patients are better matched for the potential clinical variables that might impact serum cytokine levels would define precise alterations in serum cytokine levels in different subsets of obese patients.

Further investigation could take into account the observation that inflammation-sensitive plasma proteins (ISPs) (fibrinogen, orosomucoid, α1-antitrypsin, haptoglobin, and ceruloplasmin) are associated with future weight gain [31].

## Conclusion

In conclusion, our study reinforces the idea that overweight status should not be considered a mere aesthetic concern, but rather should be adequately addressed to avoid triggering obesity and related diseases.

## Abbreviations

β-FGF: β-fibroblast growth factor; BAT: Brown adipose tissue; BM: Bone marrow; BMAT: Bone marrow adipose tissue; BMI: Body mass index; EPC: Endothelial progenitor cells; FBS: Fetal bovine serum; HS: Healthy weight sera; HSC: Hematopoietic stem cells; IL: Interleukin; MEM: Minimum essential medium; MSC: Marrow stromal cells; OS: Overweight sera; PBS: Phosphate buffered saline; RT-PCR: Reverse transcriptase-polymerase chain reaction; TNF-α: Tumor necrosis factor-α; WAT: White adipose tissue.

## Competing interests

The authors declare that they have no competing interests.

## Authors’ contributions

SC, GD and GM carried out the molecular studies. SD carried out biological assays and cell cultures with the contribution of SC and GM. MM and GP carried out the patient evaluations. FC participated in the design of the study and performed the statistical analysis. UG and MC conceived of the study, and participated in its design and coordination and helped to draft the manuscript. All authors read and approved the final manuscript.

## Supplementary Material

Additional file 1 Parameters for RT-PCR analysis. Click here for file

Additional file 2 Demographic data of enrolled patients. Click here for file

Additional file 3 **Effect of HS, OS and fetal bovine (FBS) sera on cell growth.** Cell proliferation was evaluated by a Quick Cell Proliferation Assay Kit II (Biovision). Cells were seeded in 96-well culture plates. At 1, 2, 5, 10 and 15 days post-plating, cells were collected and counted. The ratio of the total number of cells at day ‘n’ to the number of cells at day ‘n – 1’ was regarded as the cell proliferation rate. Click here for file
