# Supplementary material for: Sera of overweight people promote in vitro adipocyte differentiation of bone marrow stromal cells
Source: Stem Cell Res Ther. 2014 Jan 9;5(1):4. doi: 10.1186/scrt393 (PMC4055107; doi:10.1186/scrt393)
Supplement: Additional file 1 — Parameters for RT-PCR analysis. [file scrt393-S1.doc]

| **GENE** | **PRIMER** | **SEQUENCE** | **ANNEALING** | **AMPLICON** |
| --- | --- | --- | --- | --- |
|  |  |  | T (°C) | Lenght (bp) |
| *Adipogenic Differentiation* | | | | |
| **CEPB-β** | **1830**  **1937** | 5’-AACATGGCTGAACGCGTGT-3’  5’-TCACAGCACAGCCCGT-3’ | 60 | 108 |
| **CEPB-δ** | **980** | 5’-AGGAGCGCAAAGAAGCTAC-3’ | 55 | 134 |
|  | **1113** | 5’-CCTTAGCTGCATCAACAGGAGT-3’ |  |  |
| **PPAR-γ** | **844**  **945** | 5’-TCGACCACGTCAATCCAGAGT-3’  5’-TCGCCTTTGCTTTGGTCAG-3’ | 59 | 102 |
| **CEPB-α** | **2173** | 5’-GCCGACGGAGAGTCTTATT-3’ | 57 | 107 |
|  | **2279** | 5’-CTTGTGCATGTTGAATGTG-3’ |  |  |
| **LPL** | **413**  **519** | 5’-ATGGCTGGACGGTAACAGGAA-3’  5’-TGACAGCCAGCTCAGCACAAT-3’ | 59 | 107 |
| **ATGL** | **618**  **720** | 5’-ATCCAGGCCAATGTCTGCA-3’  5’-GGTTGTCTGAAATGCCACCAT-3’ | 62 | 103 |
|  |  |  |  |  |
|  |  |  |  |  |
|  |  |  |  |  |
| *Osteogenic Differentiation* | | | | |
| **OSTERIX** | **2803**  **2905** | 5’-TGCCTTTCCTGTAACGTTGGA-3’  5’-CCACAATGTTCTCTTCCCAAG-3’ | 58 | 103 |
| **OSTEOPONTIN** | **463** | 5’-GGTCACTGATTTTCCCACGGA-3’ | 59 | 144 |
|  | **606** | 5’-TGGATGTCAGGTCTGCGAAAC-3’ |  |  |
| *Housekeeping gene* | | | | |
| **GAPDH** | **121**  **281** | 5’-GGAGTCAACGGATTTGGTCGT-3’  5’-ACGGTGCCATGGAATTTGC-3’ | 58 | 161 |

Supplementary File 2 - Parameters for RT-PCR Analysis
