# Supplementary material for: Sera of overweight people promote in vitro adipocyte differentiation of bone marrow stromal cells
Source: Stem Cell Res Ther. 2014 Jan 9;5(1):4. doi: 10.1186/scrt393 (PMC4055107; doi:10.1186/scrt393)
Supplement: Additional file 2 — Demographic data of enrolled patients. [file scrt393-S2.doc]

Supplementary File 1

|  | | | |
| --- | --- | --- | --- |
| Blood Sample | Age | Gender | Status |
| **32** | 29 | Male | HS |
| **33** | 35 | Male | HS |
| **34** | 41 | Male | HS |
| **39** | 29 | Male | HS |
| **48** | 53 | Male | HS |
| **35** | 44 | Male | OS |
| **36** | 31 | Male | OS |
| **37** | 30 | Male | OS |
| **38** | 42 | Male | OS |
| **40** | 28 | Male | OS |
| **45** | 30 | Male | OS |
| **47** | 45 | Male | OS |
| **49** | 45 | Male | OS |
