# Supplementary figures and images for: Sera of overweight people promote in vitro adipocyte differentiation of bone marrow stromal cells
Source: Stem Cell Res Ther. 2014 Jan 9;5(1):4. doi: 10.1186/scrt393 (PMC4055107; doi:10.1186/scrt393)

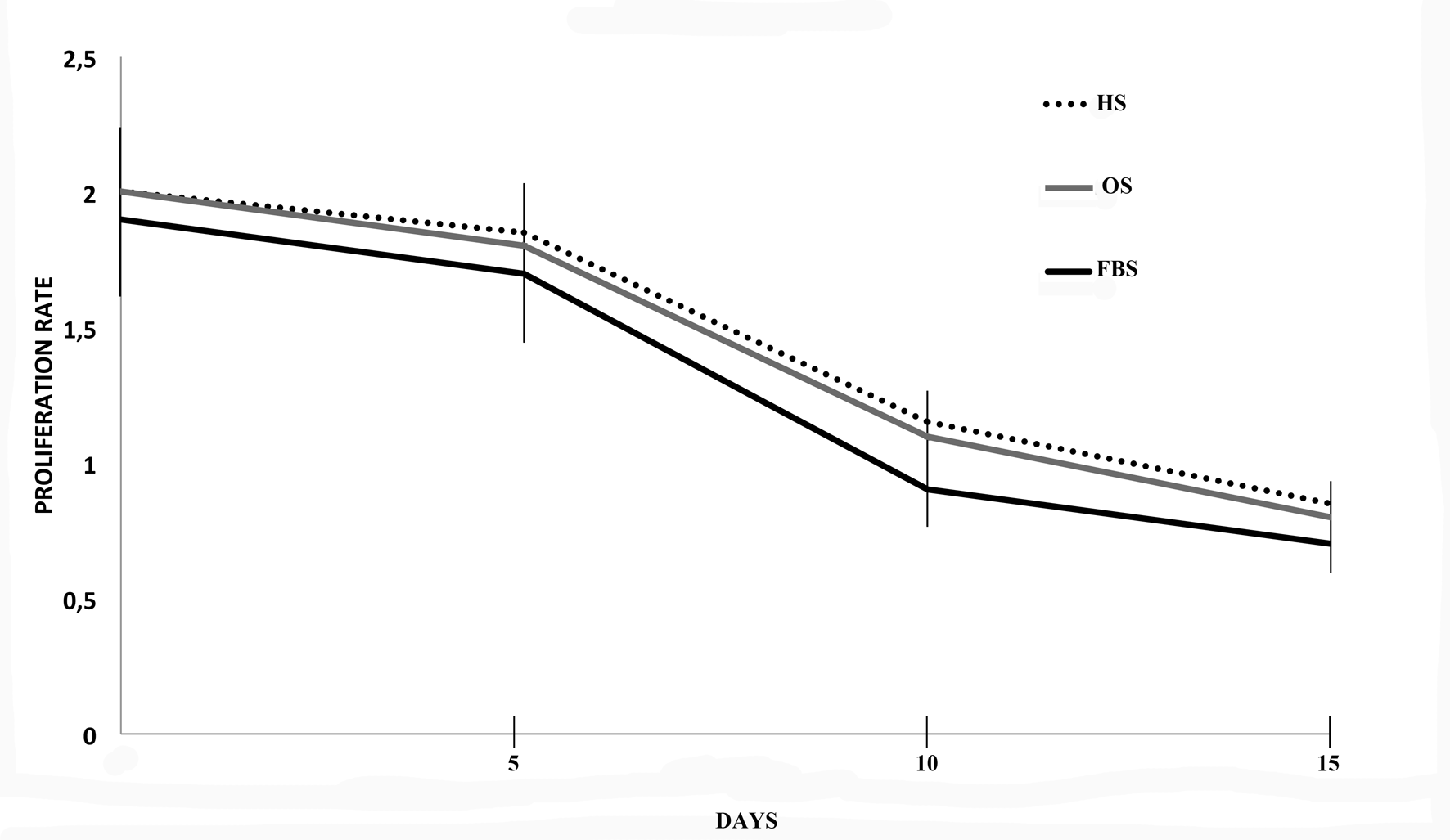

Supplement: Additional file 3 — Effect of HS, OS and fetal bovine (FBS) sera on cell growth. Cell proliferation was evaluated by a Quick Cell Proliferation Assay Kit II (Biovision). Cells were seeded in 96-well culture plates. At 1, 2, 5, 10 and 15 days post-plating, cells were collected and counted. The ratio of the total number of cells at day ‘n’ to the number of cells at day ‘n – 1’ was regarded as the cell proliferation rate. [file scrt393-S3.tiff]
